# A cell-based model to study mechanisms of endothelial-dependent thrombin generation in response to inflammation and its modulation by hydroxychloroquine

**DOI:** 10.1016/j.rpth.2024.102665

**Published:** 2024-12-25

**Authors:** Deepa J. Arachchillage, Golzar Mobayen, Mike Laffan, Anna M. Randi, Josefin Ahnström, Charis Pericleous

**Affiliations:** 1 Centre for Haematology, Department of Immunology and Inflammation, Imperial College London, London, United Kingdom; 2 Department of Haematology, Imperial College Healthcare NHS Trust, London, United Kingdom; 3 National Heart and Lung Institute, Imperial College London, London, United Kingdom

**Keywords:** coagulation, cytokines, endothelium, inflammation, thrombin generation

## Abstract

**Background:**

Inflammation is a driver of thrombosis, but the phenomenon of thromboinflammation has been defined only recently, bringing together the multiple pathways involved. *In vitro* models can support the development of new therapeutics targeting the endothelium and also assess the existing immunomodulatory drugs, such as hydroxychloroquine, in modulating the inflammation-driven endothelial prothrombotic phenotype.

**Objectives:**

To develop a model for thrombin generation (TG) on the surface of human endothelial cells (ECs) to assess pro/antithrombotic properties in response to inflammation. Furthermore, to elucidate the mechanisms of TG regulation and its modulation by immunomodulatory therapies.

**Methods:**

Cytokine-induced (tumor necrosis factor [TNF]-α, interleukin [IL]-1β, and interferon-γ) effects on ECs isolated from umbilical veins or human aortic tissue were assessed using calibrated automated thrombography in platelet-poor plasma. The expression of key coagulant and inflammatory regulators was measured at the mRNA level. Tissue factor (TF) protein levels were further assessed by flow cytometry.

**Results:**

Endothelial stimulation with TNF-α or IL-1β caused ECs to trigger TG without the addition of exogenous TF, with higher TG observed after 6 hours of stimulation than 24 hours. IL-1β induced higher peak thrombin (170 ± 5.9 nM vs 115 ± 4.9 nM), endogenous thrombin potential (1632 ± 35 nM ∗ min vs 1370 ± 23 nM ∗ min) presented as mean ± SD, and TF expression (∼2.8-fold higher) compared with TNF-α at 6 hours. Interferon-γ stimulation failed to induce TG and TF expression. The immunomodulatory drug, hydroxychloroquine, reduced cytokine-induced TG and downregulated TF expression.

**Conclusion:**

We provide detailed optimization of a robust *in vitro* model to assess the induction of an inflammation-driven endothelial prothrombotic phenotype that is also sensitive to immunomodulatory therapies, providing a tool for investigating mechanisms of disease and new drugs.

## Essentials

• Thromboinflammation brings together the multiple pathways leading to the pathogenesis of thrombosis.
• An endothelial cell model of thrombin generation was developed to assess the response to inflammation.
• Endothelial inflammation-driven prothrombotic phenotype is sensitive to immunomodulators.
• *In vitro* endothelial cell model provides a tool for investigating mechanisms of disease and drugs.

## 1 Introduction

Inflammation has long been recognized as a driver of thrombosis, but the phenomenon of thromboinflammation has been defined only recently, bringing together multiple interacting pathways [1]. Inflammation is driven by the production and release of molecular mediators, including cytokines such as tumor necrosis factor (TNF)-α, interleukin (IL)-1, IL-6, and interferons (IFNs) [2]. Inflammation upregulates the production of procoagulant plasma factors such as fibrinogen and factor (F)VIII, which are well known to increase thrombosis and were the main focus of earlier research [3]. Although the endothelium is recognized as an important regulator of hemostasis and is responsive to inflammation, its contribution to thrombosis is still only partly understood, perhaps reflecting the lack of suitable *in vitro* models [4].

The healthy endothelium presents an antithrombotic surface to flowing blood and regulates clot formation in multiple ways, including vascular contractility, platelet adhesion, coagulation, and fibrinolysis. Expression and release of endothelial nitric oxide and prostacyclin modulate vascular contractility and platelet aggregation, while anticoagulant factors expressed on the surface of the endothelium regulate the coagulation cascade. In humans, 3 natural anticoagulant pathways exist: the tissue factor (TF) pathway inhibitor (TFPI) is the main regulator of the extrinsic pathway of coagulation, while the activated protein C (APC) pathway regulates the propagation phase of coagulation by inactivating the procoagulant cofactors FVa and FVIIIa. Both the TFPI and APC pathways function in conjunction with their cofactor, protein S [5]. Finally, the antithrombin pathway acts to inhibit some of the free coagulation proteases, primarily FXa and thrombin, and also attenuate the activity of FVIIa [6]. The main source of TFPI is the endothelium, and the vast majority is bound to the endothelial surface with low levels circulating in plasma [7]. APC is generated from protein C through proteolytic activation by the thrombin-thrombomodulin (TM) complex after binding to the endothelial protein C receptor (EPCR). Both TM and EPCR are transmembrane proteins expressed on the endothelial surface [5]. While mainly expressed by hepatocytes, protein S is also expressed by endothelial cells (ECs). The endothelial surface is covered in glycosaminoglycans, including heparan sulfate, which significantly enhances antithrombin function. As such, a healthy endothelium is essential for coagulation regulation through all 3 endogenous anticoagulant pathways.

Inflammation is well known to disturb the antithrombotic features of the endothelium by upregulating prothrombotic and antifibrinolytic factors such as von Willebrand factor and plasminogen activator inhibitor-I [4]. However, inflammation also impacts the coagulation cascade directly by inducing local expression of TF, the initiator of coagulation, while simultaneously downregulating the expression of anticoagulant factors TFPI, TM, and EPCR, creating an environment favoring thrombosis [8].

An important link connecting inflammation with thrombosis is the expression of TF. Cells that constitutively express TF are not in direct contact with blood, such as vascular smooth muscle cells [9]. Upon endothelial injury and disruption of vessel wall integrity, blood comes into direct contact with TF, leading to activation of coagulation and thrombin generation (TG). Although ECs generally do not express TF in the resting state, inflammation upregulates the synthesis and expression of endothelial TF, leading to thrombus formation on the endothelial surface [10].

Reproducing the complex interplay between blood and vasculature in the regulation of thrombus formation in the laboratory is not straightforward; most studies to date have concentrated only on the contribution of plasma components without appreciation of the contribution of the endothelium. This limits the study of thrombotic disease mechanisms and the development of antithrombotic drugs that target specifically the endothelium. Therefore, a model to study endothelial-mediated coagulation and the ability of drugs to modulate the prothrombotic effects *in vitro* is needed [11].

Calibrated automated thrombography (CAT) is a commonly used method to assess TG in plasma and yields much more information than standard clotting tests, such as prothrombin and activated partial thromboplastin time. Although TG using CAT has been successful in reproducing some thrombotic disorders such as protein C or protein S deficiency, APC resistance (including FV Leiden) or the G20210A prothrombin gene mutation [[12], [13], [14], [15]], and antiphospholipid syndrome [16], it relies on an artificial procoagulant stimulus using exogenous TF at an arbitrary concentration and does not incorporate the pro- and anticoagulant properties of the endothelium.

*In vitro* TG on the surface of ECs has been performed previously [[17], [18], [19], [20], [21]]. These studies suggested that ECs influence coagulation in platelet-poor plasma (PPP) through endogenous expression of TF and anticoagulant factors. For instance, Campbell et al. [22] showed how endothelial dysfunction of human umbilical vein ECs (HUVECs) contributes to clot formation, structure, and stability. Furthermore, Billoir et al. [16] used CAT on HUVECs to demonstrate the effect of immunoglobulin (Ig) G from patients with antiphospholipid syndrome upon coagulation induced by exogenous TF. Furthermore, the effects of EC passage number, the unwanted activation of the contact system through the application of bovine calf serum, and the effects of vascular location have been described previously [23,24]. However, there has been no detailed methodological study published to date defining the assay conditions and their sensitivity to a range of cytokines, stimulation time, optimal EC density, and direct effect on pro- and anticoagulant protein expression. Developing an *in vitro* model incorporating ECs allows us to investigate mechanisms of disease so far unexplored, eventually using patient-derived ECs (endothelial colony-forming cells).

In this study, we aimed to develop a model for TG induced on the surface of ECs to assess the endothelium’s pro/anticoagulant properties in response to inflammation and elucidate the mechanisms by which TG is regulated without the addition of exogenous TF. Additionally, we assessed whether the immunomodulatory drug hydroxychloroquine (HCQ) can dampen inflammation and have a corresponding effect on inflammation-induced endothelial TG.

## 2 Methods

### 2.1 Cell culture, cytokines, and immunomodulating agents

Commercially obtained pooled HUVECs or single donor human aortic ECs (HAECs) were cultured in complete endothelial growth medium (Lonza) containing 10% fetal bovine serum (FBS; Labtech) in 96-well culture plates for CAT or 6-well plates for RNA and protein analysis (VWR International Ltd) that were previously coated with 1% gelatine for a minimum of 20 minutes. Experiments were performed at passage 4 and repeated a minimum of 3 times.

Recombinant human TNF-α, IL-1β, or IFN-γ (PeproTech), and HCQ (Merck), etanercept (Eta; TNF-α inhibitor, Enbrel, Pfizer), and anakinra (Ana; IL-1 receptor-α antagonist, Kineret, Sobi) were employed as described below.

### 2.2 Preparation of PPP

Blood samples were obtained by venipuncture from 25 healthy volunteers with no history of thrombosis, bleeding, or anti-inflammatory medication. The study was approved by the Human Research Authority and Health and Care Research Wales (reference: 22/PR/145). The first 3 mL of blood drawn after the venipuncture was discarded. Blood to be used for the plasma pool was collected into 10 mM sodium citrate (final concentration in the blood) and 18 μg CTI (Enzyme Research Laboratories) per milliliter blood (equivalent to ∼40 μg/mL CTI in plasma), and PPP was isolated as previously described [25]. Although FXII activation on ECs may play a role in physiological homeostasis and pathological thrombosis, as our aim was to provide a model to study the inflammation-induced prothrombotic phenotype driven mainly by the TF pathway, the contact pathway was inhibited. Without inhibiting the contact pathway, it is not possible to study this effect alone, as the contact pathway can be activated simply by contact with the tissue culture plate alone. Briefly, the blood was double centrifuged with a first spin at 3500 × *g* for 15 minutes without brake, followed by a second centrifugation step at 11,000 × *g* for 10 minutes. All PPP was pooled and aliquoted to 1 mL cryotubes and stored at −70 °C until assayed. PPP was defrosted in a water bath at 37 °C until they were fully thawed before being used in CAT.

### 2.3 Phospholipid preparation

Synthetic phospholipids (Avanti Polar Lipids) 1,2-dioleoyl-*sn*-glycero-3-phosphocholine, 1,2-dioleoyl-*sn*-glycero-3-phosphoserine, and 1,2-dioleoyl-*sn*-glycero-3-phosphoethanolamine were mixed in a molar ratio of 60:20:20, respectively. Unilamellar phospholipid vesicles were obtained via extrusion, as described previously [26].

### 2.4 TG using CAT

TG on the surface of ECs was recorded using CAT with a Fluoroscan Ascent FL plate reader (Thermo Labsystem) and the Thrombinoscope software (Synapse BV) as previously described [26] on confluent EC monolayers. For this, HUVECs or HAECs (0.5 × 10^4^ to 1.2 × 10^4^ per well) were seeded in 100 μL endothelial growth medium for 48 hours, followed by cytokine stimulation at 0.1 to 100 ng/mL TNF-α or IL-1β, or 1 to 1000 Units/mL IFN-γ (corresponding to 0.05-50 ng/mL) for 6 or 24 hours. These concentrations were selected in keeping with previous studies by others [27]. To confirm the specificity of cytokine-driven TF expression, Eta (160 ng/mL or 200 ng/mL) [28] or Ana (750 ng/mL and 1500 ng/mL) [29] were coincubated with TNF-α or IL-1β, respectively. To assess the effect of the immunomodulatory drug HCQ on inflammation-induced endothelial prothrombotic phenotype, ECs were pretreated with HCQ (1-10 μg/mL) for 18 hours prior to cytokine stimulation for 6 hours.

Following cytokine stimulation, ECs were washed once with 0.5% bovine serum albumin **(**BSA) in tris-buffered saline (TBS; 20 mM Tris, pH 7.4, 150 mM NaCl; 250 μL/well). TG was assessed in PPP (80 μL/well) supplemented with 4 μM phospholipid vesicles, diluted in 0.5% BSA in TBS, making a total volume of 100 μL/well. To demonstrate TG dependency on endothelial TF expression, the TG assay was performed in the presence of 15 μg/mL human anti-TF monoclonal antibodies (Mab TFE, Enzyme Research Laboratories) or class-matched IgG controls (IgG2a, κ Isotype Ctrl, BioLegend). TG was initiated by dispensation of 16.6 mM CaCl_2_ and monitored using 0.42 mmol/L of the fluorogenic substrate Z-Gly-Arg-AMC-HCl (Bachem). All concentrations are given as final in the 120 μL/well assay volume. All experiments were performed in triplicate wells and repeated a minimum of 3 times.

### 2.5 Gene expression

HUVECs were seeded into 6-well culture plates (3 × 10^5^ per well: proportionate to the number of cells used for a 96-well plate based on surface area) and treated with cytokines (0.1-100 ng/mL for TNF-α and IL-1β and 1-1000 Units/mL for IFN-γ) with or without HCQ (1-10 μg/mL) as per TG assays. Total RNA was isolated using the RNeasy Kit (Qiagen), reverse transcribed using the qScript cDNA Synthesis Kit (VWR), and quantitative real-time polymerase chain reaction (PCR) was performed using SYBR Green (Bio-Rad; QuantStudio 6 Real-Time PCR System, Thermo Fisher Scientific) using the following protocol: enzyme activation at 50 °C for 2 minutes; denaturation at 95 °C for 10 minutes; annealing and extension at 60 °C for 45 seconds (40 cycles). Seven target genes were assessed: *F3* (TF), *TFPI*, *THBD* (TM), *EPCR, PROS1* (protein S), and cell adhesion molecules *VCAM-1* and *ICAM-1* (see sequences of the primers in the Supplementary Table). For *TFPI*, a sequence shared between TFPIα and TFPIβ was used, allowing for quantification of all *TFPI* mRNA independent of the splice variant. Gene expression values were normalized to β-actin (*ACTB*). Results are presented as fold change compared with unstimulated cells. Samples were tested in triplicate in 3 independent experiments.

### 2.6 Flow cytometry

Cell surface TF expression was assessed by flow cytometry. For this, HUVECs were cultured as described above in 6-well culture plates (3 × 10^5^ per well) for 48 hours and stimulated with 10 ng/mL of TNF-α or IL-1β or 200 Units/mL of IFN-γ (equivalent 10 ng/mL) in the presence or absence of Eta or Ana for 6 hours. Following detachment, cells were washed and stained with anti-TF antibody (5 μg/mL; phycoerythrin-conjugated monoclonal, BD Biosciences) or isotype control in cell staining buffer (BioLegend) for 20 minutes in the dark at room temperature, followed by washing and fixation in 4% paraformaldehyde-phosphate-buffered saline. TF expression was analyzed by flow cytometry (BD Accuri C6 Plus, BD Biosciences), gating on a single-cell endothelial population (excluding doublets). Data were analyzed using FlowJo software v10.8 by setting unstimulated cells at 5% TF surface expression and assessing percentage positivity in stimulated cells. Unstained unstimulated controls were used in each experiment, showing no difference between TF-stained and unstained samples.

### 2.7 Statistical analysis

Results are presented as absolute values or fold change compared with untreated cells, and significant differences between groups were assessed with one-way analysis of variance or the Kruskall–Wallis test. Fold changes compared with unstimulated or untreated cells (set as 1) were assessed using the Wilcoxon test. Two groups were compared by either an unpaired *t*-test or Mann–Whitney U-test, depending on the distribution of the data. The normality of data distribution was assessed with the Shapiro–Wilk test. Descriptive statistics are expressed as mean ± SD or median and IQR. *P* < .05 was considered as statistically significant. All analyses were performed using GraphPad Prism 10.0.3.

## 3 Results

### 3.1 TG on the EC surface can be triggered by inflammatory cytokines and is cytokine-dependent

**Figure 1:**
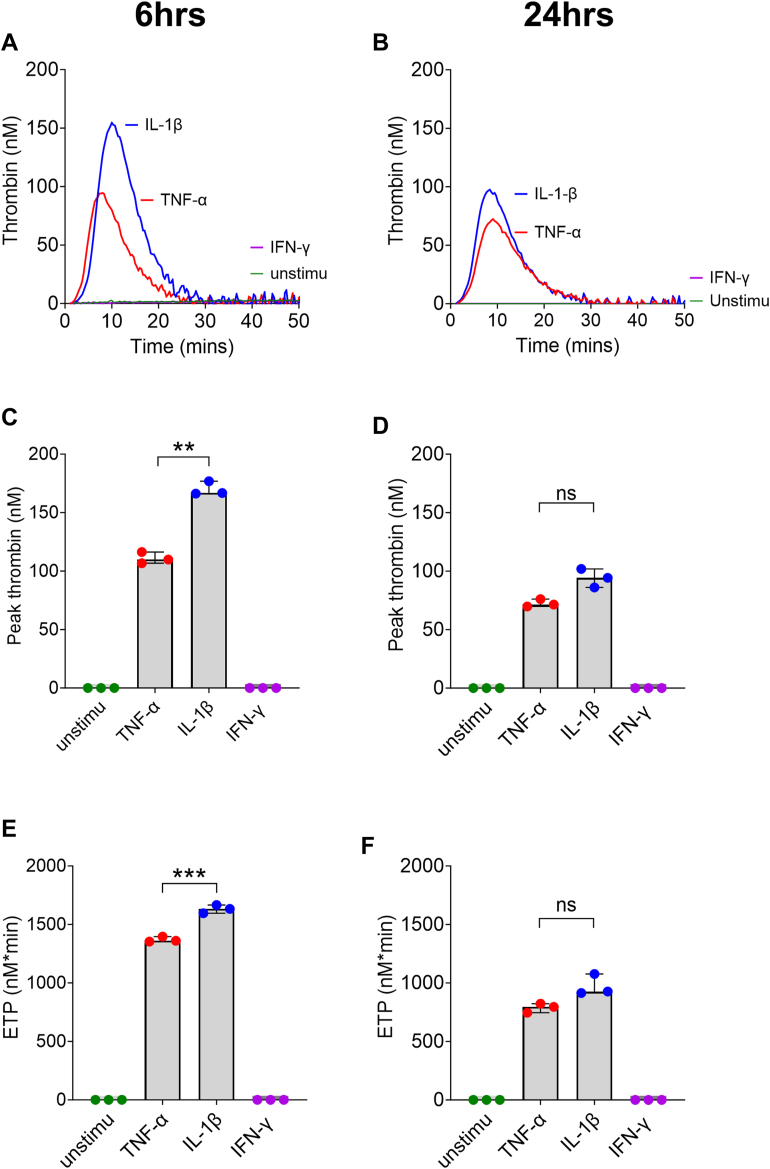
Stimulation of human umbilical vein endothelial cells (HUVECs) with tumor necrosis factor (TNF)-α, interleukin (IL)-1β, and interferon (IFN)-γ shows significantly higher thrombin generation (TG) after IL-1β treatment compared with TNF-α with no TG after IFN-γ stimulation. Pooled HUVECs were seeded at 1 × 10^4^ cells/well. Once confluent, they were treated with TNF-α or IL-1β (10 ng/mL) for 6 or 24 hours. Following treatment, TG was measured over the HUVEC monolayer in normal plasma, supplemented with 4 μM phospholipids. Representative experiments (*n* = 4) obtained from cells stimulated for either 6 hours (A) or 24 hours (B) are shown. Peak thrombin (C) and endogenous thrombin potential (ETP; E) at 6 hours and peak thrombin (D) and ETP (F) at 24 hours with TNF-α and IL-1β are shown and presented as median and IQR (*n* = 3). ∗∗*P* < .01, ∗∗∗*P* < .001 according to the Mann–Whitney U-test. Ns, not significant; Unstimu, unstimulated.

To investigate the effect of inflammation on EC procoagulant activity, we seeded HUVECs (1 × 10^4^ cells/well) and allowed these to grow into a confluent monolayer in 96-well plates. To model an inflammatory state, confluent cells were stimulated with IL-1β (10 ng/mL), TNF-α (10 ng/mL), or IFN-γ (200 Units/mL) for 6 or 24 hours. Following stimulation, CAT was performed over the cell surface to test whether the stimulated cells could induce TG in PPP. Importantly, no exogenous initiator of coagulation was added to these assays, and thrombin began with recalcification of the TG well with fluorogenic substrate and CaCl_2_. Thrombin was readily generated in wells containing IL-1β- or TNF-α–stimulated cells, whereas no thrombin was generated over unstimulated cells or cells stimulated with IFN-γ (Figure 1A, B). The TG over TNF-α–stimulated cells is in agreement with previous studies [30]. Intriguingly, higher TG was achieved over cells incubated with TNF-α for 6 hours compared with 24 hours (Figure 1C, D), with peak heights of 115 (IQR, 9.6) nM and 73.9 (IQR, 6.3) nM, respectively (*P* = .02). The same was observed after IL-1β treatment with peak heights of 170 (IQR, 10.6) nM and 94.3 (IQR, 15.9) nM being generated after 6 and 24 hours of stimulation, respectively (*P* = .01; Figure 1C, D). We also found that peak thrombin and endogenous thrombin potential (ETP) were significantly higher after 6 hours of stimulation with IL-1β compared with TNF-α (Figure 1C, E). In contrast, no significant difference was observed after 24 hours (Figure 1D, F).

**Figure 2:**
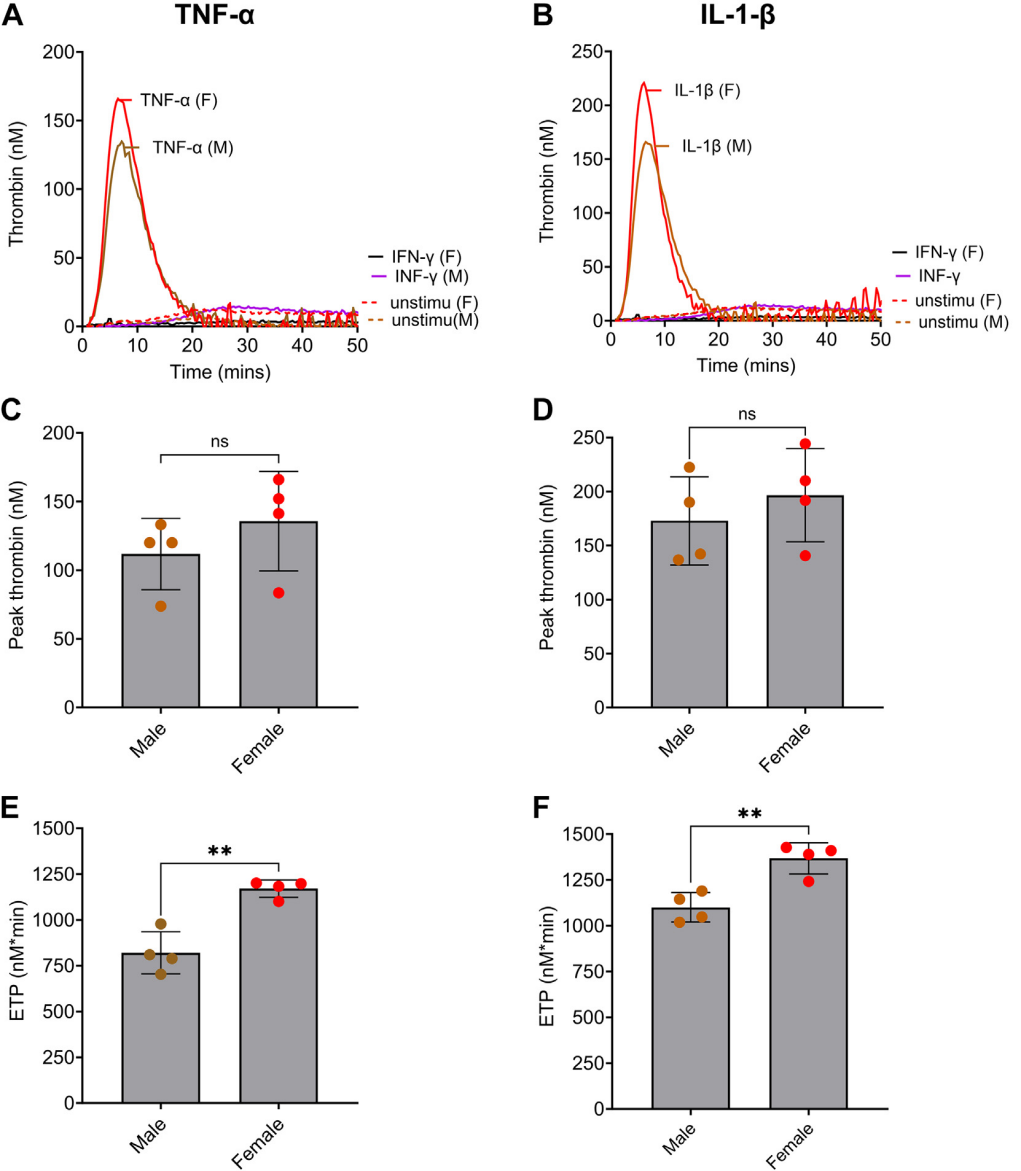
Thrombin generation on the surface of human aortic endothelial cells (HAECs) following stimulation with tumor necrosis factor (TNF)-α, interleukin (IL)-1β, or interferon (IFN)-γ for 6 hours. HAECs from male (M) or female (F) donors were seeded at 1 × 10^4^ cells/well. Once confluent (∼48 hours), they were treated with TNF-α, IL-1β (10 ng/mL), or IFN-γ (10 Units/mL) for 6 hours. After treatment, thrombin generation was measured in normal plasma, supplemented with 4 μM phospholipids, over the HAEC monolayer. Representative experiments (*n* = 4) obtained from cells stimulated with either TNF-α (10 ng/mL) or IFN-γ (10 Units/mL; A) or IL-1β (10 ng/mL) or IFN-γ (10 Units/mL) at 6 hours (B) are shown. Peak thrombin after stimulating with TNF-α (C) and IL-1β (D) and endogenous thrombin potential (ETP) after stimulating with TNF-α (E) and IL-1β (F) are plotted as median and IQR (*n* = 4). ∗∗*P* < .01 according to the Mann–Whitney U-test. Ns, not significant; Unstimu, unstimulated.

To confirm that the procoagulant effect was not specific to HUVECs, the TG assays were also performed on HAECs from male or female donors. As in the experiments described above, HAECs were stimulated by TNF-α (Figure 2A, C, E), IL-1β (Figure 2B, D, F), or IFN-γ (Figure 2A, B) for 6 hours. Similar to our observations of HUVECs, stimulation with TNF-α and IL-1β promoted TG on HAECs, but stimulation by IFN-γ failed to generate thrombin (Figure 2A, B). When comparing male vs female HAEC donors, a significantly higher ETP (Figure 2E, F) was observed in female vs male HAECs following stimulation with both IL-1β and TNF-α. Peak thrombin showed a similar but nonsignificant trend (Figure 2C, D).

### 3.2 Endothelial TG is driven by endogenous TF expression

**Figure 3:**
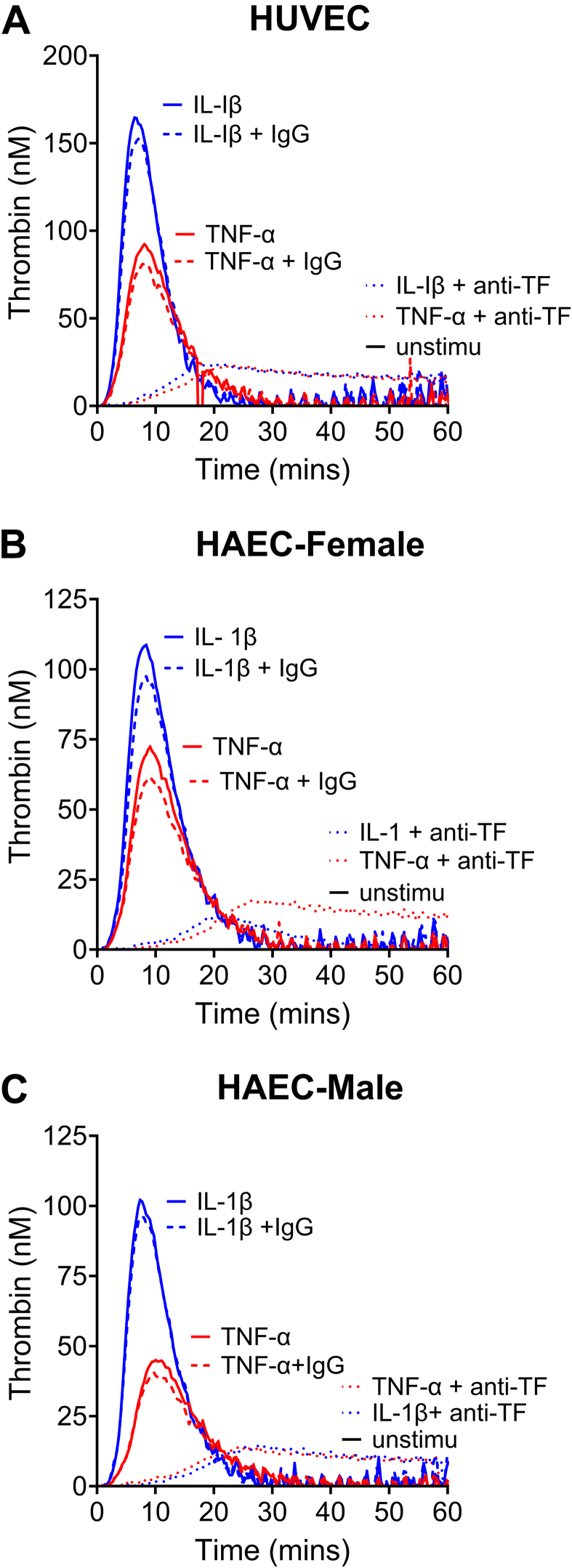
Endothelial thrombin generation following stimulation with cytokines is driven by endogenous tissue factor expression. Human umbilical vein endothelial cells (HUVECs) and human aortic endothelial cells (HAECs) were seeded at 1 × 10^4^ cells/well. Once confluent (∼48 hours), they were treated with tumor necrosis factor (TNF)-α, interleukin (IL)-1β (10 ng/mL), or interferon (IFN)-γ (10 Units/mL) for 6 hours. After washing, thrombin generation was measured over the endothelial monolayer in the presence of 4 μM phospholipids and the presence or absence of inhibitory monoclonal antitissue factor (TF) antibodies (Mab TFE; Enzyme Research Laboratories) in parallel with a class-matched immunoglobulin (Ig) G control. Representative data are shown for HUVECs (A), HAECs from female donors (B), and HAECs from male donors (C) of *n* = 4. Unstimu, unstimulated.

From the CAT assays described above, it is clear that stimulating ECs with TNF-α or IL-1β can cause TG in PPP. However, these experiments alone do not pinpoint the molecular trigger for the reaction. The use of corn trypsin inhibitor (CTI)-treated plasma and the absence of exogenous TF in the TG reaction suggested dependency on an endogenous initiator that is generated by EC stimulation. Since EC stimulation has been shown to induce TF expression [30], we repeated the assays on HUVECs (Figure 3A) and also with male and female HAECs (Figure 3B, C) in the presence and absence of inhibitory anti-TF antibodies. For both cell types, the inhibitory anti-TF antibodies significantly reduced the TG induced by exposure to TNF-a and IL-1β (10 ng/mL), while no effect was seen with a class-matched IgG control. These experiments confirmed that cytokine-induced TG on the EC surface is due to endogenous TF expression. Importantly, since the procoagulant response and TF dependency in HAECs were similar to that observed in HUVECs, these results also show that HUVECs can be used as a reliable model for assessing the role of the endothelium in inducing TG. Thus, HUVECs were used for all subsequent experiments.

### 3.3 Testing the effect of different cell densities and cytokine doses on TG

Following the demonstration that ECs can trigger TG via endogenous TF expression, we determined the optimal cell number to be seeded into 96-well plates while keeping all other variables constant (timing of seeding and culture prior to stimulation and cytokine dose). While statistically significantly lower and slower TG was seen at the lowest number (0.5 × 10^4^ cells/well) after stimulation with both TNF-α and IL-1β for 6 hours (Supplementary Figures S1 and S2, respectively), no differences in TG parameters were seen between 0.8 and 1.2 × 10^4^ cells/well. Thus, all subsequent experiments were performed with 1.0 × 10^4^ cells/well as the midpoint.

**Figure 4:**
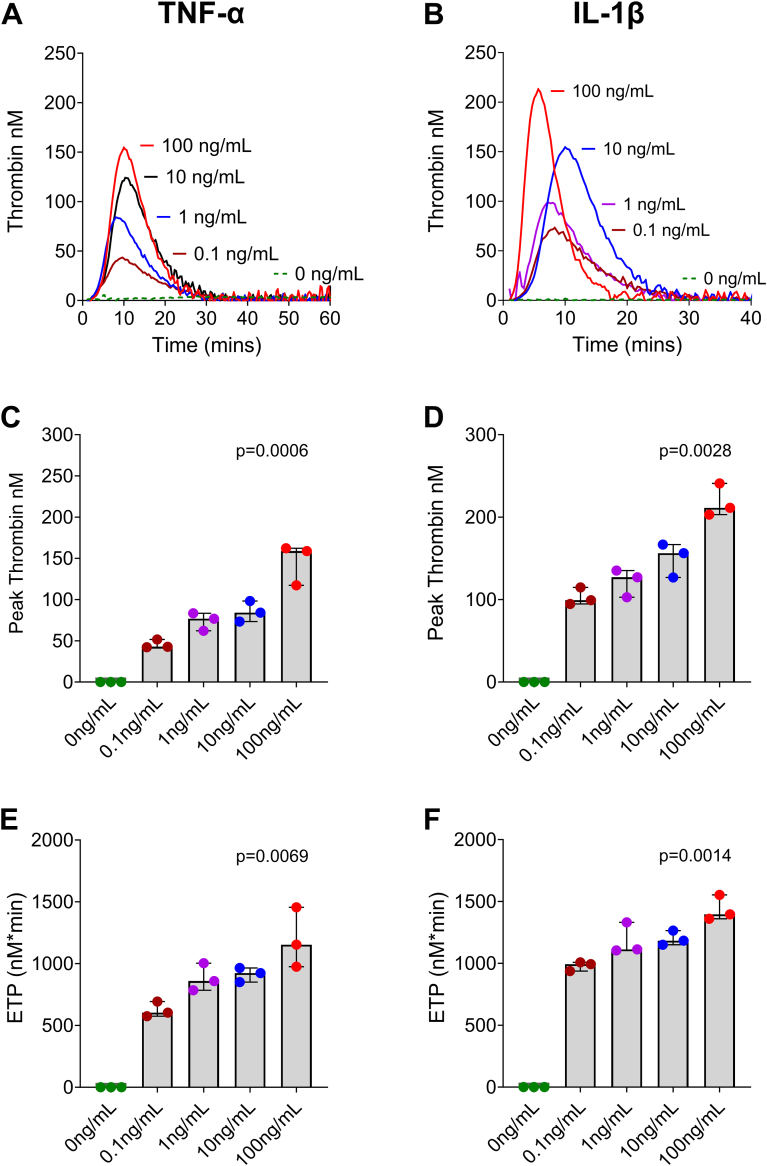
Inflammatory cytokines dose-dependently increase endothelial thrombin generation (TG). Human umbilical vein endothelial cells were stimulated with increasing concentrations (0.1-100 ng/mL) of tumor necrosis factor (TNF)-α (A, C, E) and interleukin (IL)-1β (B, D, F) for 6 hours, followed by measurement of TG in normal plasma and followed over time. Representative TG curves (A and B) obtained from cells stimulated by TNF-α (A) and IL-1β (B) are shown for *n* = 3. Peak thrombin (C and D) and endogenous thrombin potential (ETP; E and F) are shown and presented as median and IQR (*n* = 3). A Kruskal–Wallis test was performed to test for the association between increasing cytokine concentration and increased TG. The *P* values are presented in each panel.

Next, cytokine dose responses were determined. Here, cells were stimulated with increasing concentrations (0-100 ng/mL) of TNF-α, IL-1β, and IFN-γ for 6 hours, followed by TG assessment (Figure 4). Once again, stimulation of cells with IFN-γ did not induce TG at any concentration tested (data not shown). In contrast, stimulation with both TNF-α and IL-1β dose-dependently increased TG, with TG observed even at the lowest cytokine concentration tested (0.1 ng/mL) and the highest TG generated after stimulation with 100 ng/mL. There were no differences in lag times between the 4 concentrations of TNF-α and IL-1β studied, suggesting saturation at the lowest concentration (0.1 ng/mL; Supplementary Figure S3A, B), with time to peak being significantly shorter with increasing concentration of TNF-α and IL-1β and the shortest time to peak at 100 ng/mL (Supplementary Figure S3C, D).

### 3.4 Coagulant-anticoagulant and adhesion molecule expression are altered by cytokine stimulation

**Figure 5:**
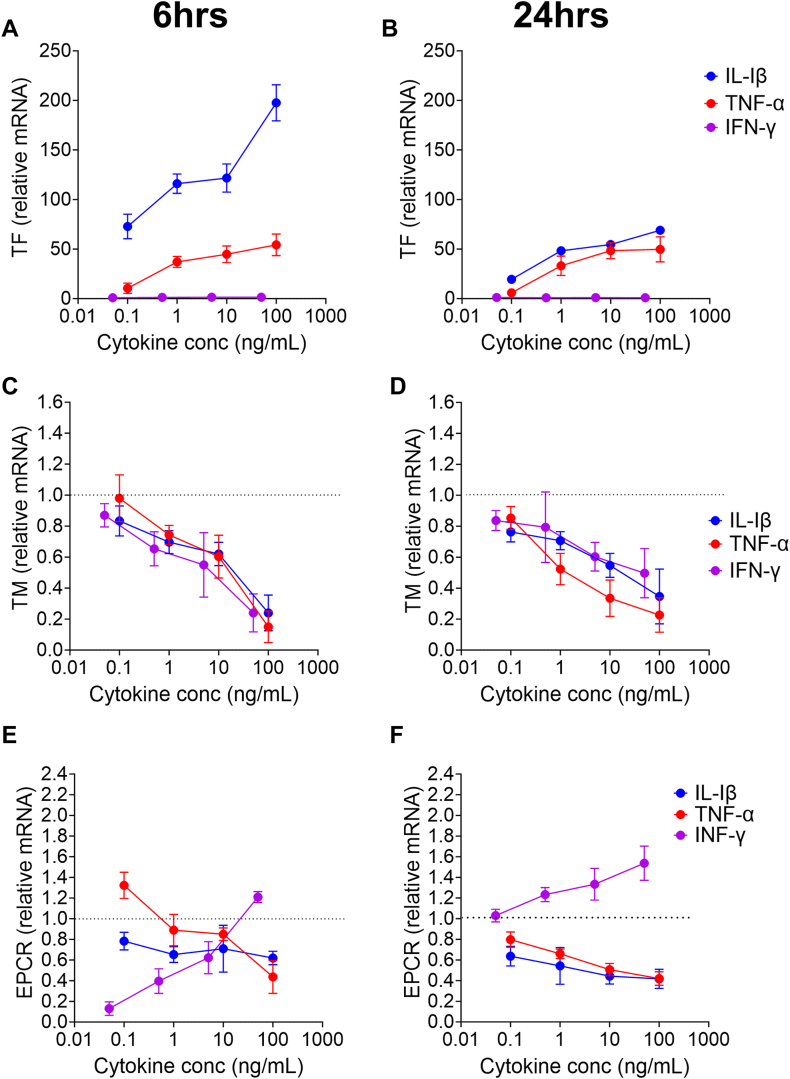
Endothelial cell mRNA expression of procoagulant and anticoagulant proteins following stimulation with cytokine at 6 and 24 hours. Human umbilical vein endothelial cells were seeded at 3 × 10^5^ cells/well (6-well culture plates) and treated with cytokines (0.1-100 ng/mL for tumor necrosis factor [TNF]-α and interleukin [IL]-1β and 0.5-50 ng/mL [1-1000 Units/mL] for interferon [IFN]-γ) with or without hydroxychloroquine (1-10 μg/mL) for 6 hours. Total RNA was isolated using the RNeasy Kit (Qiagen), reverse transcribed using the qScript cDNA Synthesis Kit (VWR), and quantitative real-time polymerase chain reaction was performed using SYBR Green (Bio-Rad; QuantStudio 6 Real-Time PCR System, Thermo Fisher Scientific). Results are presented as fold change compared with unstimulated cells. Samples were tested in triplicate in 3 independent experiments. EPCR, endothelial protein C receptor; TM, thrombin-thrombomodulin.

With the CAT assays showing that TG is triggered by TF after cytokine stimulation, we next determined whether the same cytokine stimulation influences EC expression of adhesion molecules as well as pro- and anticoagulant factors. As expected, stimulation of HUVECs with all 3 cytokines dose-dependently increased both VCAM-1 (Supplementary Figure S4A, B) and ICAM-1 (Supplementary Figure S4C, D) mRNA. Critically, for the TG experiments, a dose-dependent increase in TF mRNA was observed following stimulation with TNF-α and IL-1β, which for IL-1β was significantly higher at 6 hours compared with 24 hours. Similarly, IFN-γ failed to upregulate TF in keeping with the TG results (Figure 5A, B). Of the anticoagulant factors, TM, EPCR, and TFPI expression were downregulated with increasing concentrations of TNF-α and IL-1β at 6 hours and 24 hours, in agreement with the increased TG (Figure 5C–F and Supplementary Figure S5A, B). The response from IFN-γ varied between the various anticoagulant proteins. Low-dose IFN-γ suppressed EPCR expression at 6 hours and dose-dependently increased expression at 24 hours (Figure 5E, F). In contrast to the other anticoagulants, the effect of cytokine stimulation on protein S mRNA levels was less uniform. Protein S was downregulated at all TNF-α and IL-1β concentrations after 6 hours of stimulation, but at 24 hours, only IL-1β dose-dependently reduced protein S expression. Similar to EPCR, low-dose IFN-γ suppressed protein S expression at 6 hours and dose-dependently increased expression at 24 hours (Supplementary Figure S5C, D).

### 3.5 Upregulation of surface TF expression in response to inflammatory cytokines is abrogated in the presence of cytokine-blocking agents

**Figure 6:**
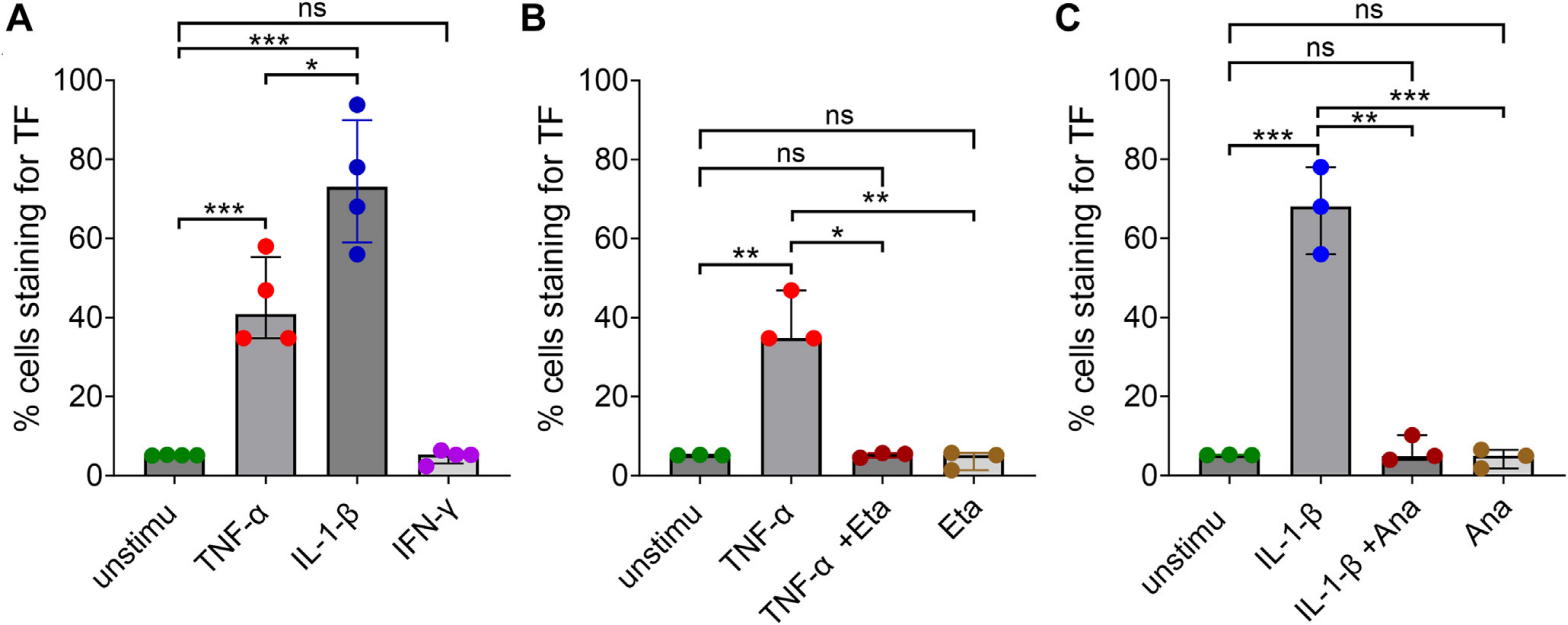
Tissue factor (TF) protein expression induction following stimulation of human umbilical vein endothelial cells with tumor necrosis factor (TNF)-α and interleukin (IL)-1β compared with unstimulated (unstimu) controls and interferon (IFN)-γ. Human umbilical vein endothelial cells were seeded in 6-well culture plates (3 × 10^5^ per well) for 48 hours and stimulated with 10 ng/mL of IFN-γ, TNF-α, or IL-1β (A) and in the presence or absence of etanercept (Eta; B) or anakinra (Ana; C) for 6 hours. Following detachment, cells were washed and stained with anti-TF antibody (5 μg/mL; phycoerythrin-conjugated monoclonal, BD Biosciences) or isotype control in cell staining buffer (BioLegend). TF expression was analyzed by flow cytometry (BD Accuri C6 Plus, BD Biosciences). Unstained unstimu controls were used in each experiment. Medians and IQRs are plotted (*n* = 3). ∗*P* < .05, ∗∗*P* < .01, ∗∗∗*P* <0.001 according to the Mann–Whitney U-test and Kruskall–Wallis test. Ns, not significant.

With TF being critical as the initiator of TG in these assays, cytokine-mediated TF induction was further quantified at the protein level using flow cytometry (Figure 6A). As expected, a significantly increased percentage of TF-positive cells was observed following exposure to 10 ng/mL TNF-α (median, 40.8%) or IL-1β (median, 73%) for 6 hours compared with 5.1% of unstimulated cells. Importantly, there was a significantly higher proportion of TF-positive cells stimulated with IL-1β compared with TNF-α, which is in agreement with the increased TF mRNA levels and increased TG in these cells. No increase after treatment with IFN-γ was noted, which is also in line with the TG and quantitative PCR results.

**Figure 7:**
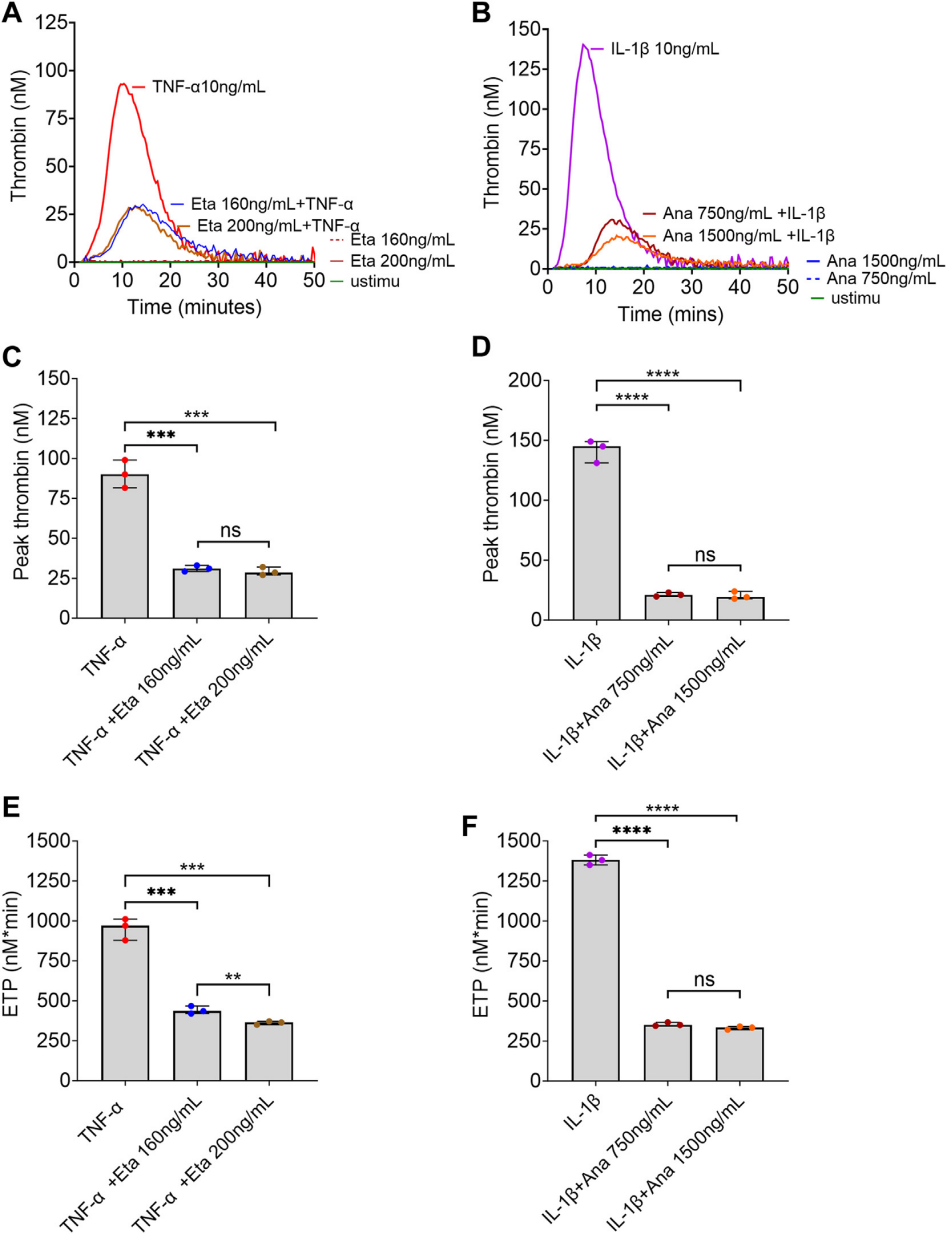
Endothelial prothrombic phenotype induced by cytokines in the presence and absence of targeted cytokine inhibitor drugs. Human umbilical vein endothelial cells were seeded at 1 × 10^4^ cells/well in 96-well culture plates. Once confluent (∼48 hours), they were treated with tumor necrosis factor (TNF)-α (10 ng/mL) in the presence or absence of etanercept (Eta; 160 or 200 ng/mL; TNF-α inhibitor) or interleukin (IL)-1β (10 ng/mL) with or without anakinra (Ana; 750 or 1500 ng/mL; IL-1 receptor-α antagonist) for 6 hours. Once washed, thrombin generation was measured in plasma supplemented with 4 μM phospholipids. Representative figures of thrombin generation (A and B) following treatment with TNF-α in the presence or absence of Eta (A) and IL-1β with or without Ana (B) are shown (*n* = 3-4). Peak heights (C and D) and endogenous thrombin potential (ETP; E and F) are presented as median and IQR (*n* = 3-4). ∗∗∗*P* < .001, ∗∗∗∗*P* < .0001 according to the Mann–Whitney U-test. Unstimu, unstimulated.

Next, we introduced cytokine-blocking agents (biologics) to ensure that the increase in TF-positive cells was indeed a direct response to cytokine stimulation. For this purpose, cells were incubated with TNF-α (10 ng/mL) in the presence and absence of Eta (160 ng/mL or 200 ng/mL) or with IL-1β (10 ng/mL) in the presence and absence of Ana (750 ng/mL or 1500 ng/mL), targeted TNF-α and IL-1β inhibitors, respectively (Figure 6B, C). While having no effect on their own, as expected, the addition of the specific blocking agent limited the proportion of TF-positive cells to levels similar to that in unstimulated cells. To confirm whether the reduction in TF-positive cells after treatment with cytokine-blocking drugs had a direct effect on TG, we performed CAT over cells treated as described above (Figure 7). As expected from the reduced proportion of TF-positive cells, both therapeutics efficiently reduced TG, with a ∼65.6% to 67.6% and 85.0% to 85.7% reduction in peak thrombin by Eta and Ana, respectively (Figure 7C, D).

### 3.6 The immunomodulatory drug HCQ modulates the inflammation-induced prothrombotic phenotype on ECs

**Figure 8:**
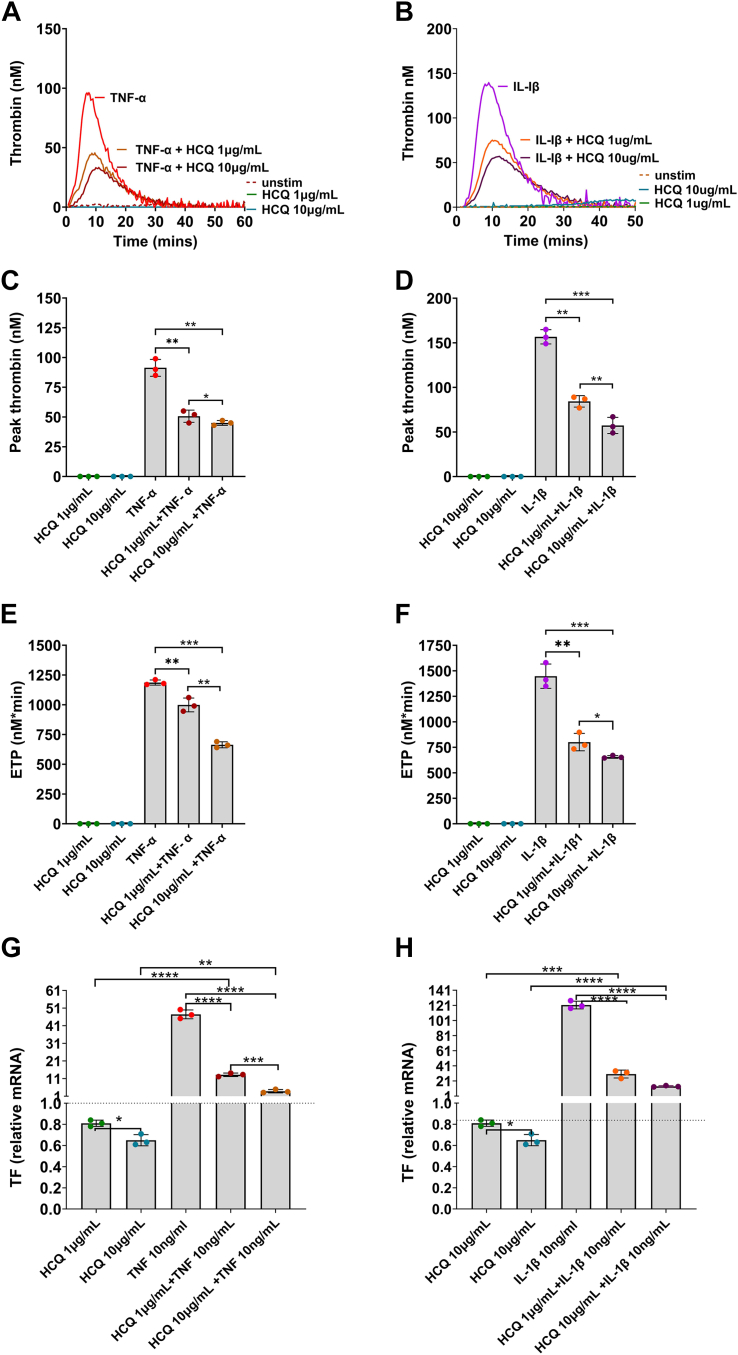
Effect of hydroxychloroquine (HCQ) on endothelial-induced thrombin generation (TG) and tissue factor (TF) expression in response to inflammation. TG was performed following pretreatment of human umbilical vein endothelial cells with 1 μg/mL or 10 μg/mL HCQ for 18 hours prior to tumor necrosis factor (TNF)-α or interleukin (IL)-1β stimulation (at 10 ng/mL for 6 hours). Representative TG curves are shown for cells treated with TNF-α (A) or IL-1β (B) in the presence and absence of HCQ (*n* = 3). Peak thrombin (C and D) and endogenous thrombin potential (ETP; E and F) are presented as median and IQR (*n* = 3). TF mRNA was determined by quantitative polymerase chain reaction using SYBR Green (Bio-Rad; QuantStudio 6 Real-Time PCR System, Thermo Fisher Scientific). Note that the scale of the y-axis is different between (G) and (H). The results are presented as mean ± SD of fold change compared with unstimulated (unstim) cells (dotted line set at 1; *n* = 3). ∗*P* < .05, ∗∗*P* < .01, ∗∗∗*P* < .001, ∗∗∗∗*P* < .0001 according to an unpaired *t*-test.

HCQ is an immunomodulatory drug used as a first-line treatment for patients with systemic lupus erythematosus and other autoimmune diseases. Some research also suggests that HCQ has antithrombotic properties, such as reduced endothelial and monocyte TF expression and upregulation of endothelial TM expression [31]. Therefore, we assessed the effects of HCQ in modulating the endothelial prothrombotic phenotype induced by inflammatory cytokines. For this purpose, we pretreated HUVECs with 1 μg/mL or 10 μg/mL HCQ for 18 hours prior to TNF-α or IL-1β stimulation (at 10 ng/mL). We found that this resulted in a significant dose-dependent reduction in TG at both concentrations of HCQ tested (Figure 8A–F). In agreement with the demonstrated mechanism above, both concentrations of HCQ downregulated TF mRNA expression induced by TNF-α or IL-1β (Figure 8G, H).

## 4 Discussion

It is well known that ECs play a crucial role in maintaining an anticoagulant environment in the vasculature. Despite this, much of coagulation research is performed in their absence. The importance of combining endothelial and coagulation research has been highlighted by the recently growing interest in thromboinflammation, where the link between endothelial inflammatory responses and thrombosis is evident. However, the research into the mechanisms involved in specific diseases is hampered by the lack of suitable *in vitro* models. In this study, we developed a cell-based model to study the prothrombotic effects of inflammatory cytokines on primary human ECs. Focusing on TNF-α, IL-1β, and IFN-γ, we demonstrated that TNF-α and IL-1β induced upregulation of endothelial TF expression at both mRNA and protein levels. It is worth noting that the data suggest an increase in TF production and surface expression rather than decryption of existing surface TF, although a contribution from this is not excluded since 5% of unstimulated ECs express TF at baseline without noticeable TG. Induction of surface TF expression resulted in spontaneous TG from both venous (HUVECs) and arterial ECs (HAECs). Importantly, our assays were performed in the absence of an exogenous TF trigger and in the presence of the FXIIa inhibitor, CTI, while also confirming endogenous TF dependency using inhibitory anti-TF antibodies.

Our data show that cytokines differ in their ability to induce an endothelial prothrombotic phenotype *in vitro*. Increased peak thrombin, ETP, and TF expression were observed following stimulation by IL-1β compared with TNF-α, while IFN-γ failed to upregulate TF or initiate TG. This highlights the importance of choosing the appropriate cytokine with care when assessing the proinflammatory response of the endothelium, in particular when mimicking specific disease states because cytokine responses and signatures differ across different chronic inflammatory and autoimmune diseases. Furthermore, our data show that the cytokine effect followed a clear time course and dose response, which is clinically relevant as thrombotic events often occur at the time of acute exacerbations and elevated cytokine levels [32]. In the future, it would be relevant to study the effect of prolonged exposure to low-dose cytokines as encountered in chronic inflammatory diseases. The concentration of cytokines used in this study spans the range seen in acute inflammatory responses, such as in patients with severe COVID-19, and the concentrations used in previous *in vitro* studies but also extend to suprapathological levels [33].

While the trigger for TG is TF expression, we further characterized the endothelial-cytokine response, noting several other changes that contribute to the thrombotic phenotype and may facilitate TG. These include the downregulation of natural anticoagulants present on the EC surface (TFPI, TM, and EPCR) mediated by TNF-α and IL-1β, as demonstrated in both acute and chronic inflammation [34,35], and variation of protein S and upregulation of endothelial adhesive molecule expression. These effects were either not seen or were less prominent with IFN-γ. Of potential interest, IFN-γ dose-dependently increased expression of EPCR following 24 hours of stimulation, which may contribute to a reduced prothrombotic phenotype. However, IFN-γ also downregulated TM as much as the other cytokines. Since all cytokines studied here may be present simultaneously during acute inflammation, IFN-γ may be able to augment TG *in vivo* even if it cannot initiate it. Following on from this study, it will be important to examine combinations of cytokines and chronic exposure as they must occur *in vivo* in diseases such as systemic lupus erythematosus, rheumatoid arthritis, or atherosclerosis to look for synergistic or antagonistic effects and how cytokine inhibitors/immunomodulatory drugs modulate the chronic inflammatory endothelial prothrombotic phenotype.

Recent evidence supports the concept of a dual role of IFN-γ in inflammation by regulating other cytokines, including expression of anti-inflammatory molecules such as IL-1 receptor antagonist and IL-18 binding protein, modulation of proinflammatory cytokine production, and activation of apoptosis in addition to upregulation of proinflammatory IL-12 and TNF-α [36]. However, as our current study’s focus was to develop a cell-based model of TG and assess the prothrombotic properties of these 3 cytokines, we did not study the above effects.

Importantly, we demonstrated that immunomodulatory treatments, such as Eta and Ana, used as disease-modifying antirheumatic drugs (DMARDs) in chronic inflammatory diseases, can downregulate cytokine-induced TG. Lack of complete inhibition following treatment with Eta and TNF-α is likely due to the concentration of TNF-α used (10 ng/mL), which is the standard concentration used in *in vitro* models of EC activation [33] but high compared with *in vivo* levels during acute inflammation, reported to reach ∼1 ng/mL [37]. However, with the inherent thrombotic risk associated with autoimmune rheumatic diseases where DMARDs are the cornerstone of treatment, cytokine inhibitors may have additional beneficial effects in preventing vascular events. In fact, recent studies have highlighted the potential of both TNF-α and IL-1β as therapeutic targets for the prevention of cardiovascular diseases [[38], [39], [40]]. Similarly, HCQ, which is also a DMARD, has a parallel antithrombotic effect by downregulating TF expression at clinically relevant concentrations (1 μg/mL) [41]. HCQ blood levels in patients with systemic lupus erythematosus are reported in the range of 0.5 to 2 μg/mL with a maximum HCQ dose of 400 mg daily [41]. Here, we used 1 and 10 μg/mL of HCQ. Although 10 μg/mL is much higher than the plasma concentration observed in patients on HCQ, the drug has a half-life of more than 40 days [42], while our treatment duration was only 18 hours prior to cytokine stimulation. Our observations with HCQ are also clinically translatable. In a previous small study of 22 patients with antiphospholipid antibodies treated with HCQ 200 mg daily, soluble TF levels were significantly reduced after 3 months of HCQ treatment compared with baseline [43].

As previously described by Snow et al. [21], we found that HAECs exposed to proinflammatory stimuli express endogenous TF and can support TG in CAT. As in our experiments using HUVECs, we found that HAECs induced TG after being stimulated by TNF-α and IL-1β but not by IFN-γ. This not only shows that both HUVECs and HAECs can be involved in triggering (or downregulating) TG but also that the mechanisms by which this occurs are similar. We further noted a significant difference in the overall thrombin generated measured by ETP in female HAECs compared with male HAECs, although there was no difference in peak thrombin generated. The observed difference in the ETP may have clinical relevance and could reflect an epigenetic effect of estrogen on the endothelium, which may contribute to a prothrombotic state in females [44]. Estrogen also influences inflammation, increasing proinflammatory cytokines such as IL-1β and TNF-α [45,46]. Although no detailed donor information was available, the age range of 4 female HAEC donors tested was 36 to 51 years old, suggesting a potentially relevant effect of estrogen. Thrombosis demonstrates vascular-bed specificity despite the fact that all vascular beds are exposed to the same blood. This model can be adapted to include ECs from different vascular beds, allowing investigation of tissue-specific mechanisms of thrombosis. Variation in shear may be another important modulator of prothrombotic inflammatory responses in different tissues, although technically difficult to incorporate [33,47].

Here, we have optimized an *in vitro* assay, assessing the impact that cytokines have on the pro- and anticoagulant properties of ECs. This is not the first time that TG has been assessed on ECs [[16], [17], [18], [19], [20], [21]]. However, while components of this study have been reported previously, this is the first methodological paper showing the importance of optimization of cell density, the concentration and incubation time of cytokine stimulation, and the choice of cytokine. Furthermore, we investigated the mechanisms responsible for the effect and showed how the model can be directly used to evaluate the thrombotic/antithrombotic phenotype of inflammatory stimuli or anti-inflammatory drugs (as highlighted by our experiments using DMARDs) on ECs. As TG has been used to assess the effectiveness of anticoagulant or procoagulant drugs [[48], [49], [50], [51]], this model can also be used to assess whether drugs have effects on the endothelium in the presence or absence of inflammation. Along the same lines, future work will incorporate platelets and immune cells, including neutrophils and monocytes, to further increase the physiological and pathological relevance of the model.

## Funding

D.J.A. is funded by the Medical Research Council UK (MR/V037633/1), and infrastructure support for some of the work was provided by the National Institute for Health and Care Research (NIHR) Imperial Biomedical Research Centre (BRC). C.P. was a recipient of Versus Arthritis Career Development and Imperial College – Wellcome Trust Institutional Strategic Support Fund fellowships.
