# Supplementary material for: A cell-based model to study mechanisms of endothelial-dependent thrombin generation in response to inflammation and its modulation by hydroxychloroquine
Source: Res Pract Thromb Haemost. 2024 Dec 25;9(1):102665. doi: 10.1016/j.rpth.2024.102665 (PMC11772944; doi:10.1016/j.rpth.2024.102665)
Supplement: Supplementary Figures [file mmc1.docx]

**Supplementary Results**


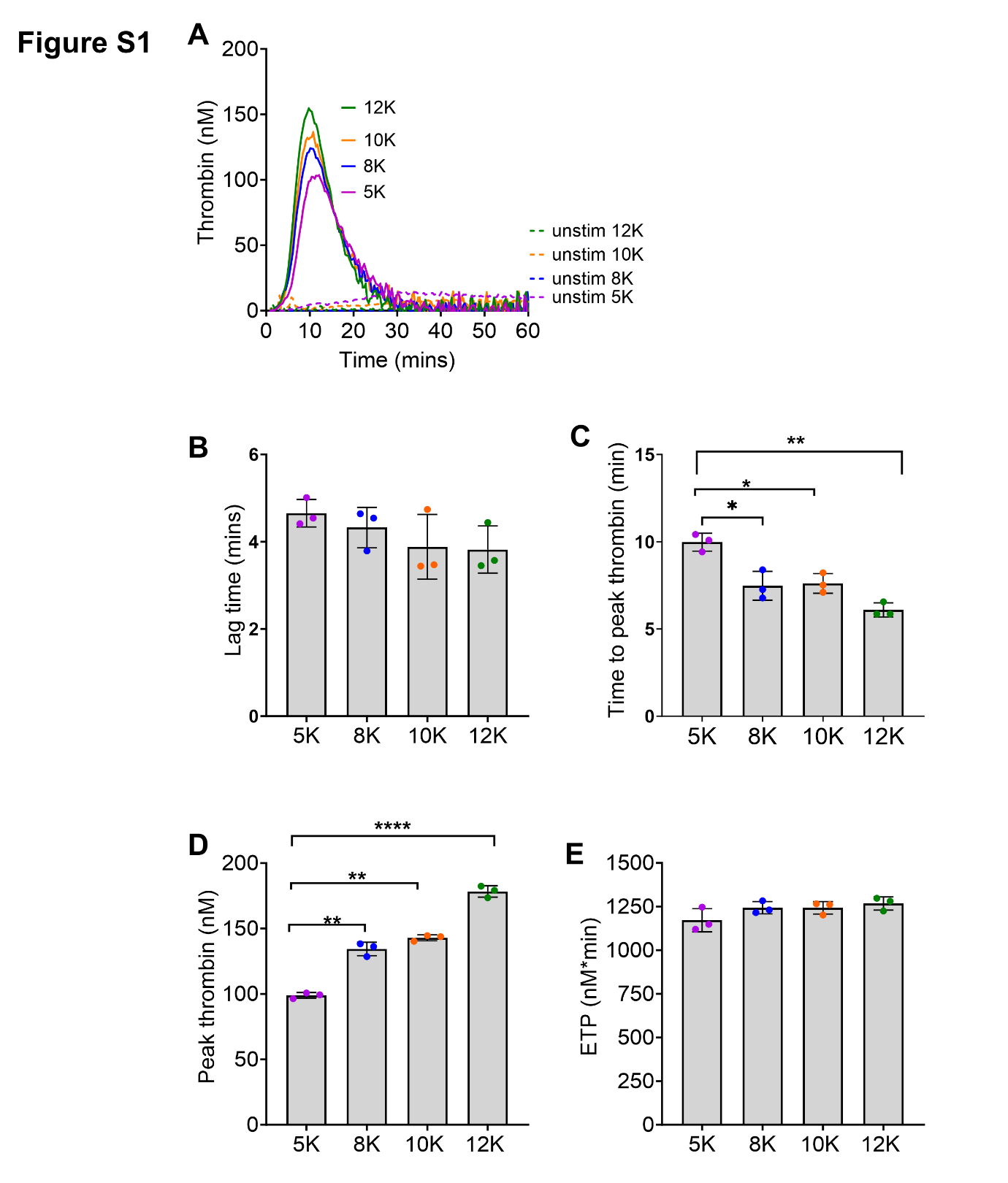


**Figure S1. Thrombin generation following 6hrs stimulation with TNF-α with increasing endothelial cell counts.**

HUVEC were seeded at 0.5x10^4^ - 1.2x10^4^ per well in 200μL EGM2 for 48hrs followed by stimulation by TNF-α (10ng/mL) for 6 hours. After washing, TG was measured over the HUVEC monolayer. For this, normal plasma was supplemented with 4μM phospholipids and TG was measured in real time. (**A**) A representative TG curve is shown (n=3). Lag time (**B**), time to peak (**C**) peak thrombin (**D**) and ETP (**E**) are shown and presented as median and IQR (n=3). *p<0.05, **p<0.01, ****p<0.0001 according to Mann Whitney test.


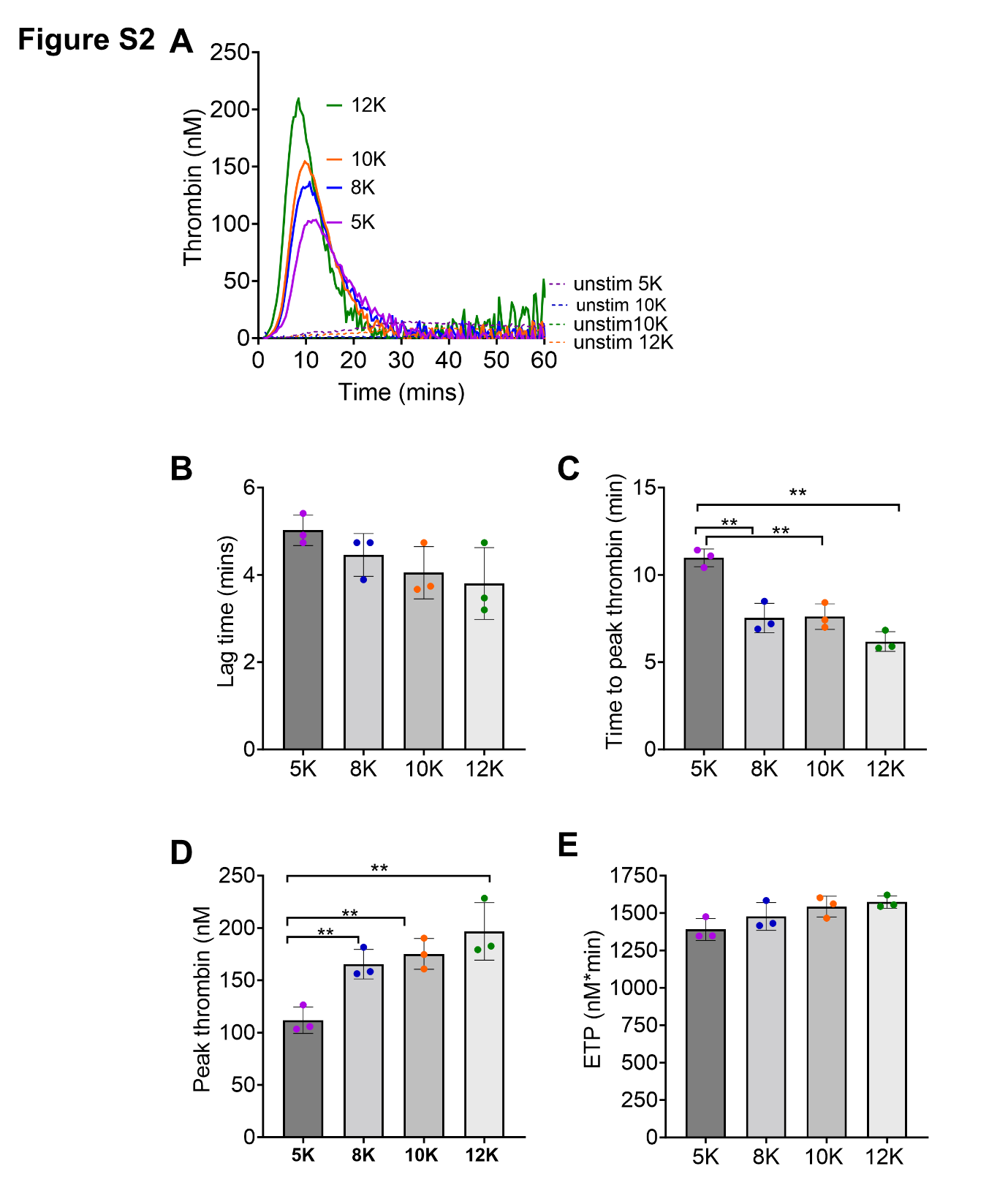


**Figure S2. Thrombin generation following 6hrs stimulation with IL-1β with increasing endothelial cell counts.**

HUVEC were seeded at 0.5x10^4^ - 1.2x10^4^ per well in 200μL EGM2 for 48hrs followed by stimulation by IL-1β (10ng/mL) for 6 hours. After washing, TG was measured over the HUVEC monolayer. For this, normal plasma was supplemented with 4μM phospholipids and TG was followed using a fluorogenic substrate. (**A**) A representative thrombin generation curve is shown (n=3). Lag time (**B**), time to peak (**C**) peak thrombin (**D**) and ETP (**E**) are shown and presented as median and IQR (n=3). **p<0.01 according to Mann Whitney test.


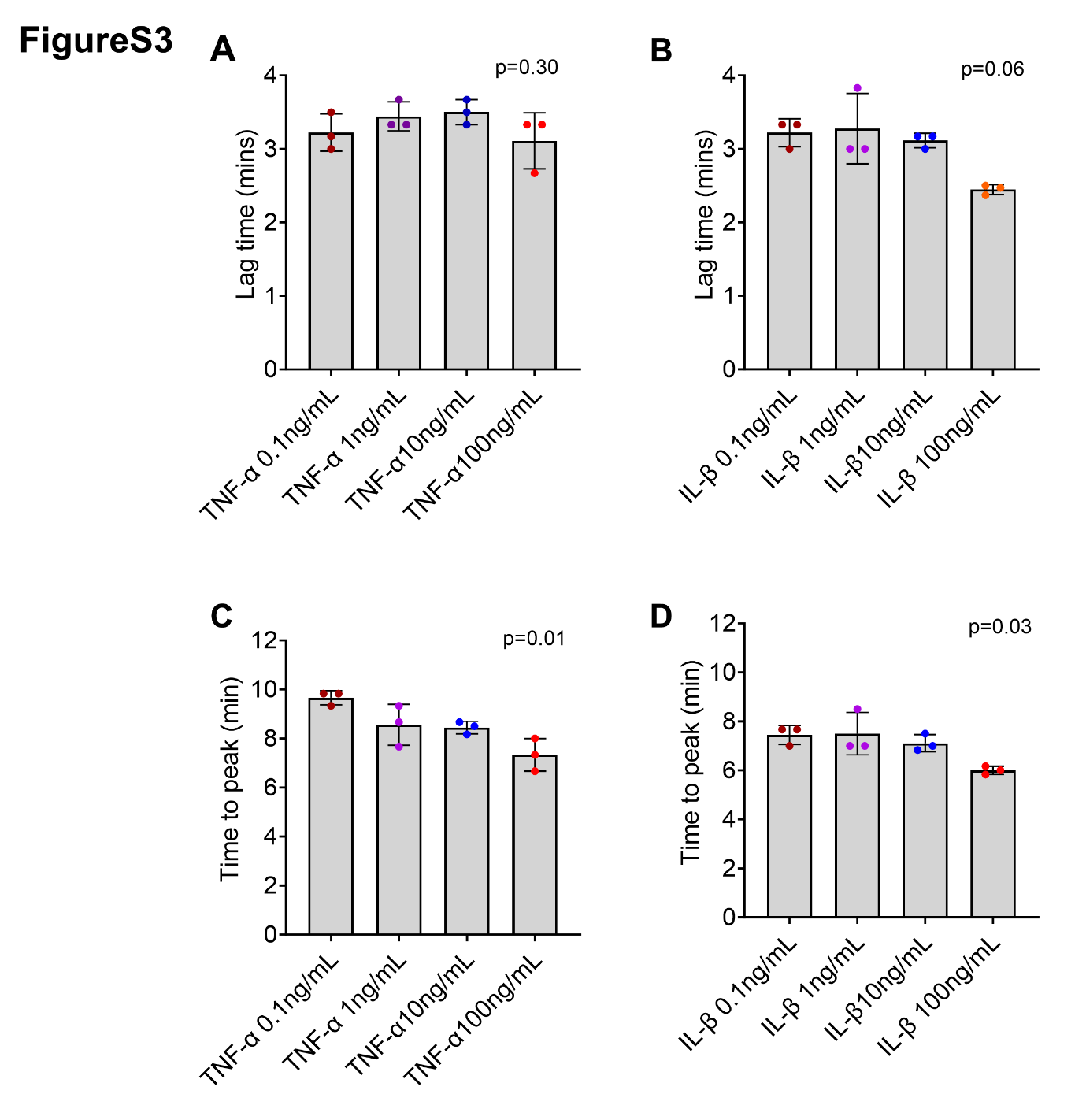


**Figure S3. Lag time and time to peak thrombin generation with increasing concentration of TNF-α and IL-1β.**

HUVEC were stimulated with increasing concentrations (0.1-100ng/mL) of TNF-α (**A, C**) and IL-1β (**B, D**) for 6 hrs, followed by measurement of TG. Normal plasma was supplemented with 4μM phospholipids and TG was followed over time. Lag times (**A-B**) and time to peak (**C-D**) are shown and presented as median and IQR (n=3). A Kruskal Wallis test was performed to test for association between increasing cytokine concentration and increased TG. The p-values are presented in each panel.


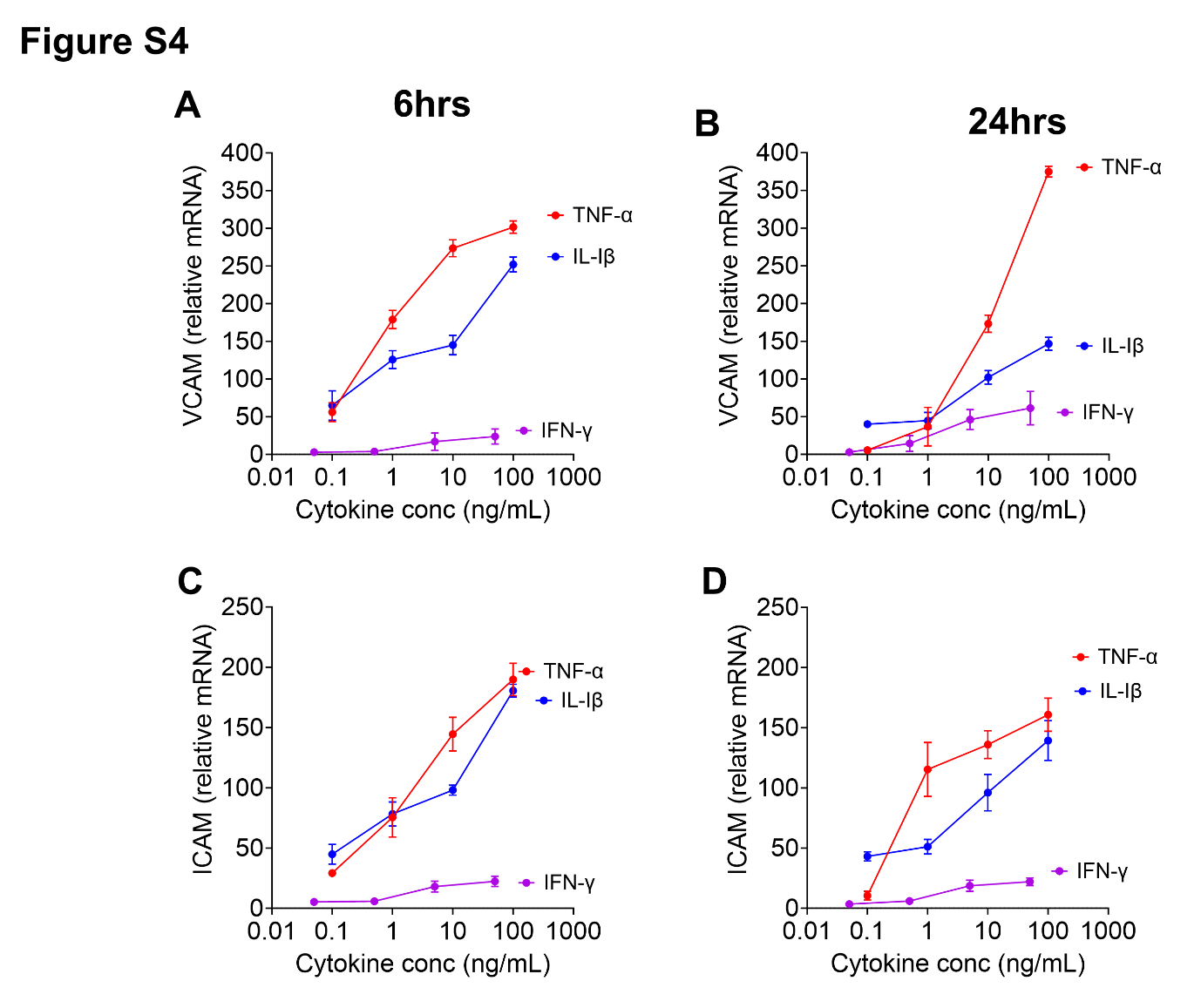


**Figure S4. Endothelial cell mRNA expression of adhesion molecules following stimulation with cytokine at 6 hrs and 24hrs.**

HUVEC were seeded at 2.5x10^5^ cells/well (6-well culture plates) and treated with cytokines (0.1-100ng/mL for TNF-α, IL-1β and 1-1000units/mL for IFN-γ) with or without HCQ (1-10µg/mL) for 6hrs. Total RNA was isolated, reverse transcribed and quantitative real-time PCR was performed. Results are presented as fold change compared to unstimulated cells. Samples were tested in triplicate in 3 independent experiments. Gene expression of VCAM at 6hrs, 24hrs (**A & B**) and ICAM at 6hrs, 24hrs (**C & D**) are shown and presented as median and IQR (n=3).

HUVEC = human umbilical vein endothelial cells, VCAM= Vascular Cell Adhesion Molecule 1, ICAM= Intracellular Adhesion Molecule


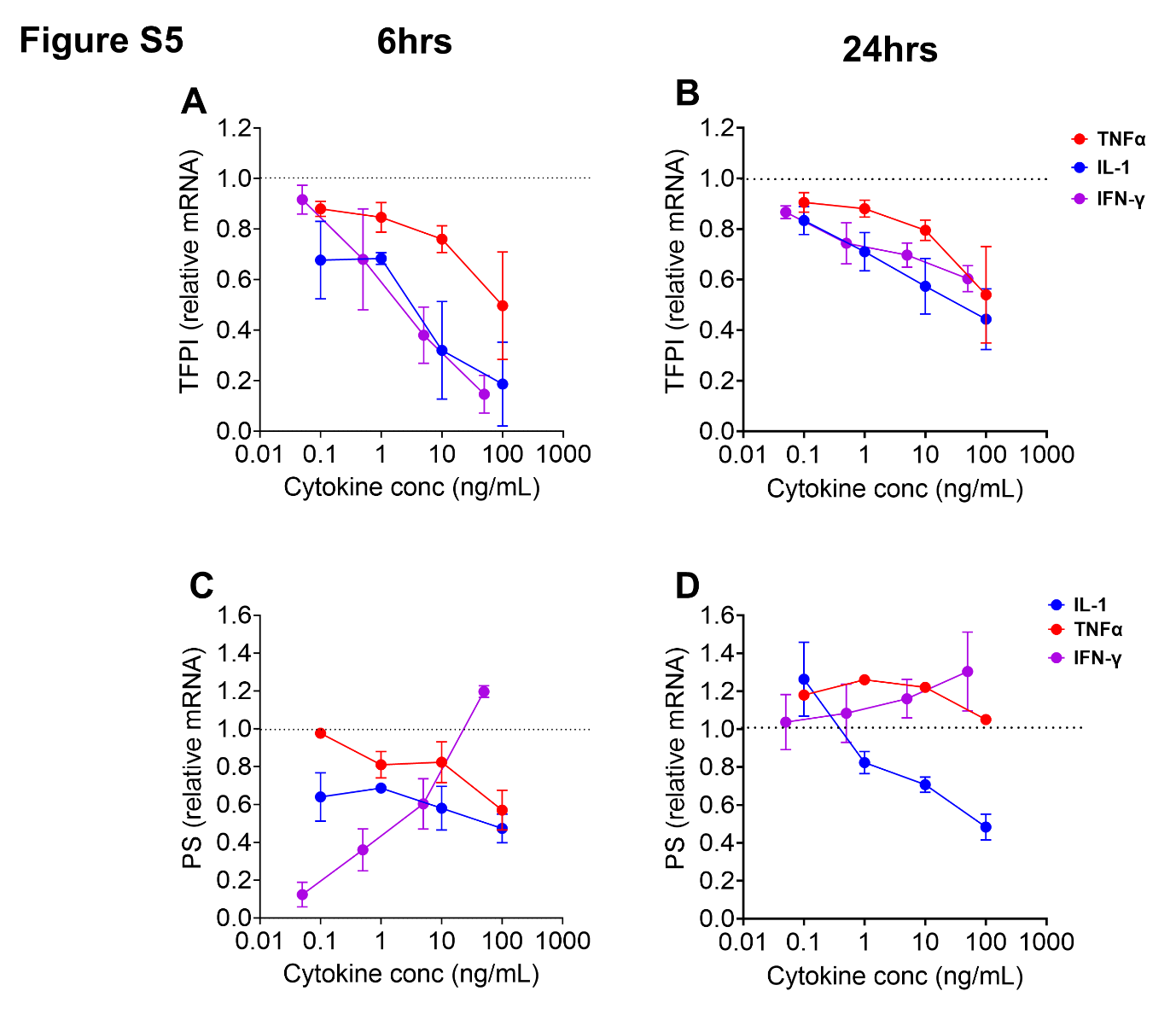


**Figure S5.** **Endothelial cell mRNA expression of anticoagulant proteins following stimulation with cytokine at 6 hrs and 24hrs.**

HUVEC were seeded at 2.5x10^5^ cells/well (6-well culture plates) and treated with cytokines (0.1-100ng/mL for TNF-α, IL-1β and 1-1000units/mL for IFN-γ) with or without HCQ (1-10µg/mL) for 6hrs. Total RNA was isolated, reverse transcribed, and quantitative real-time PCR was performed. Results are presented as fold change compared to unstimulated cells. Samples were tested in triplicate in 3 independent experiments. Gene expression of TFPI at 6hrs, 24hrs (A &B) and PS at 6hrs, 24hrs (C &D) are shown and presented as median and IQR (n=3).TFPI= Tissue Factor Pathway Inhibitor, PS=Protein S

**Table S1. List of primers used in qPCR.**

| **Gene** | **FWD primer** | **REV primer** | **Source** |
| --- | --- | --- | --- |
| TM | AACGACCTCTGCGAGCACTTCT | CCAGTATGCAGTCATCCACGTC | Origene, USA |
| TFPI | CAGCTCAATGCTGTGAATAACTCC | TCTGCTGGAGTGAGACACCATG |  |
| VCAM-1 | GATTCTGTGCCCACAGTAAGGC | TGGTCACAGAGCCACCTTCTTG |  |
| ICAM-1 | AGCGGCTGACGTGTGCAGTAAT | TCTGAGACCTCTGGCTTCGTCA |  |
| TF | CAGAGTTCACACCTTACCTGGAG | GTTGTTCCTTCTGACTAAAGTCCG |  |
| EPCR | GCTCAATGCCTACAACCGCACT | CGAAGTGTAGGAGCGGCTTGTT |  |
| Protein S | GGCTCCTACTATCCTGGTTCTG | CAAGGCAAGCATAACACCAGTGC |  |
| ACTB | CACCATTGGCAATGAGCGGTTC | AGGTCTTTGCGGATGTCCACGT |  |

FWD – forward, REV – reverse.

TM= thrombomodulin, TFPI= tissue factor pathway inhibitor, VCAM = Vascular Cell Adhesion Molecule, ICAM = Intracellular Adhesion Molecule, TF= Tissue factor, ECPR = Endothelial protein C receptor, ACTB= β-actin
